# Use of Prescription Opioids and Initiation of Fatal 2-Vehicle Crashes

**DOI:** 10.1001/jamanetworkopen.2018.8081

**Published:** 2019-02-15

**Authors:** Stanford Chihuri, Guohua Li

**Affiliations:** 1 Center for Injury Epidemiology and Prevention, Columbia University Medical Center, New York, New York; 2 Department of Anesthesiology, College of Physicians and Surgeons, Columbia University, New York, New York; 3 Department of Epidemiology, Mailman School of Public Health, Columbia University, New York, New York

## Abstract

**Importance:**

The prevalence of prescription opioids detected in fatally injured drivers has increased markedly in the past 2 decades in the United States. It is unclear whether driver use of prescription opioids plays a role in fatal crash causation.

**Objective:**

To assess the association between driver use of prescription opioids and the risk of being culpable of crash initiation in fatal 2-vehicle crashes.

**Design, Setting, and Participants:**

This pair-matched study was based on data from the Fatality Analysis Reporting System for drivers involved in fatal 2-vehicle crashes on US public roads between January 1, 1993, and December 31, 2016. Data analysis was conducted from December 8, 2017, to December 7, 2018.

**Exposures:**

Testing positive for prescription opioids compared with testing negative, and blood alcohol concentrations (BACs) based on toxicological testing results.

**Main Outcomes and Measures:**

Culpability of crash initiation and adjusted odds ratios and 95% CIs.

**Results:**

A total of 36 642 drivers involved in 18 321 fatal 2-vehicle crashes were included. The most common driving error leading to fatal 2-vehicle crashes was failure to keep in lane (7535 [41%]). Drivers culpable of initiating the crashes were more likely than their nonculpable counterparts to test positive for prescription opioids (918 [5.0%] vs 549 [3.0%]; *P* < .001), alcohol (BAC ≥0.01 g/dL, 5258 [28.7%] vs 1815 [9.9%]; *P* < .001), and both substances (1.0% vs 0.3%, *P* < .001). The adjusted odds ratio of crash initiation was 2.18 (95% CI, 1.91-2.48) for drivers testing positive for prescription opioids compared with drivers testing negative, and increased with BACs (BAC 0.01-0.07 g/dL: adjusted odds ratio, 1.97; 95% CI, 1.75-2.22; BAC ≥0.08 g/dL: adjusted odds ratio, 8.20; 95% CI, 7.42-9.07; compared with BAC <0.01 g/dL). There was no significant interaction effect on crash initiation between prescription opioid use and alcohol use.

**Conclusions and Relevance:**

Driver use of prescription opioids was associated with initiation of 2-vehicle crashes, independent of alcohol use. Clinicians should take into consideration the adverse effect of opioid analgesics on driving safety while prescribing these medications and counseling patients.

## Key Points

**Question:**

In fatal 2-vehicle crashes, is driver use of prescription opioids associated with increased risk of being culpable of initiating the crashes?

**Findings:**

In this study of 36 642 drivers involved in 18 321 fatal 2-vehicle crashes, prescription opioid use as indicated by toxicological testing results was associated with a significantly increased risk of crash initiation, due in large part to failure to keep in proper lane.

**Meaning:**

Use of prescription opioids by drivers is increasingly implicated as a contributory cause in fatal motor vehicle crashes.

## Teaser

This pair-matched study assesses whether driver use of prescription opioids is associated with crash initiation in fatal 2-vehicle crashes.

## Introduction

Driving under the influence of drugs is a public health concern in the United States and around the world.^1,2,3,4,5^ In the United States, motor vehicle crashes are the second leading cause of unintentional injury deaths after drug overdose.^6^ Reversing the longstanding downward trend, death rates per 100 million vehicle miles traveled increased 2.6% from years 2015 to 2016.^7^ Concern about drugged driving has heightened in recent years due in part to increasing permissibility and availability of marijuana, excess consumption of prescription opioids,^5,8,9,10^ and highly publicized driving under the influence of drugs–related crashes.^11,12^ Opioid use may result in dizziness, drowsiness, and sedation, which may impair the requisite psychomotor and cognitive skills necessary for safe driving.^4,5,13,14,15^ In addition, opioid use may also impair concentration and attention, decrease alertness, and increase reaction time.

In October 2017, the US federal government declared the opioid epidemic a national public health emergency due to the unabated increase in overdose mortality. In 2016, 11.5 million people aged 12 years or older reported that they had misused prescription opioids in the previous year.^16^ Among patients who receive prescription opioids for chronic pain, 21% to 29% misuse them and 8% to 12% develop an opioid use disorder.^17^ Although the overall opioid prescription rate per 100 persons has declined from 72.4% in 2006 to 66.5% in 2016, prescription rates remain high at more than 214 million total annual opioid prescriptions.^16^ About 25% of US counties dispense opioid prescriptions enough for their entire respective populations and in some counties prescription rates for opioids are up to 7 times the national rate.^16,18^ The prevalence of prescription opioids detected in drivers who died within 1 hour of crash increased from 1% in 1995 to 7.2% in 2015.^15^ Hydrocodone, oxycodone, and morphine are the most commonly detected prescription opioids among fatally injured drivers.^15^ Few epidemiological studies have assessed the role of prescription opioids in fatal motor vehicle crash initiation.

Recent reviews of experimental and epidemiological studies have found that driver use of prescription opioids is associated with increased cognitive impairment and crash risk.^14,19,20,21,22^ Nonetheless, studies assessing the risk of opioid use on crash initiation have produced inconsistent results,^23,24,25,26,27^ mainly owing to the increasing prevalence of prescription opioids detected in the general driver population and in fatally injured drivers and differences in research designs and analytical approaches across studies.^14^ In the present study, we apply a pair-matched design to a large sample of drivers involved in fatal 2-vehicle crashes to assess the role of prescription opioid use in the initiation of fatal 2-vehicle crashes. The pair-matched design allows us to compare the 2 drivers involved in the same crash based on initiation status while minimizing confounding associated with spatiotemporal and environmental characteristics (eg, weather, road conditions, time of day, day of the week, seasonality, traffic regulations, and law enforcement), thus leading to potentially more accurate estimates of crash initiation risk associated with prescription opioid use than alternative approaches.

## Methods

### Data Source

Data for this study came from the Fatality Analysis Reporting System (FARS) compiled and maintained by the National Center for Statistics of the National Highway Traffic Safety Administration. The FARS is a repository of detailed data on all motor vehicle crashes that occur on public roads in all 50 states, the District of Columbia, and Puerto Rico and that result in at least 1 fatality (either a vehicle occupant or a nonmotorist) within 30 days of the crash.^28^ Data are acquired from various documents such as coroner or medical examiner records, state vehicle registration and licensing files, police reports, death certificates, medical records, and vital statistics.^28,29^ Trained analysts collect, code, and record the information into the FARS database using standardized forms and procedure. Data elements are organized into person, vehicle, and crash levels.^29^ Person-level data include characteristics of people involved in the crash, such as sex, age, survival status, driving history within the previous 3 years, role in the crash, and drug and alcohol toxicological testing results. Vehicle-level data include information about each of the involved vehicles, such as vehicle type, model, make, year, weight rating, and driving errors or harmful events that led to the crash. Crash-level data include crash circumstances, such as manner of collision, weather conditions, roadway type, date and time, location, and number of vehicles and people involved.^28,29,30^ Passenger vehicle type was identified based on the vehicle body type classification and gross vehicle weight rating. Several quality assurance programs, including checks for completeness, consistency, timeliness and accuracy are used to continuously monitor the FARS data.^28^

Crash responsibility was assigned based on driver-related factors obtained from the narrative of police reports and other supporting materials. For each crash, the FARS analysts recorded up to 3 (years 1993-1996) or 4 (years 1997-2016) driver-related factors.^28^ Driver-related factors (codes 17-60) are often referred to as unsafe driver actions or driving errors, which have contributed to crash initiation.^27,29^ In the present study, for each 2-vehicle crash, the driver with 1 or more driving errors that led to the crash was treated as crash initiator, whereas the other driver with no errors was treated as noninitiator. The use of driving errors as a proxy measure of crash culpability is preferred to traffic violations because traffic violations may require legal proof and are not uniformly recorded.^31^ In addition, the coding of driving errors has been associated with the physical configuration of the crash and has been widely used in epidemiologic studies.^27,31,32,33,34,35,36^

### Study Design and Study Participants

**Figure 1.**
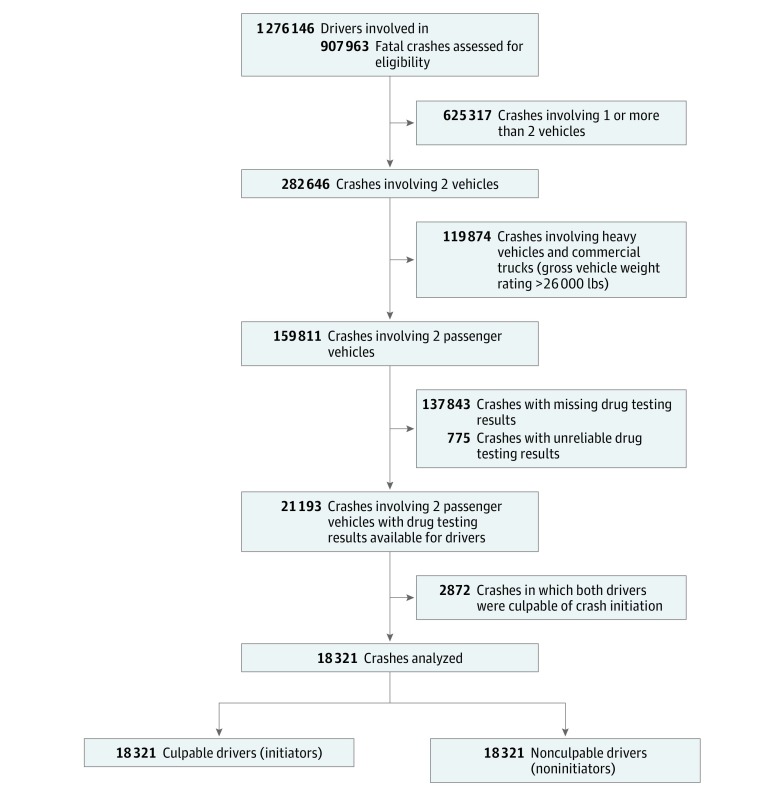
Flowchart of Selection of Drivers Involved in Fatal 2-Vehicle Crashes, Fatality Analysis Reporting System, 1993-2016

The study sample consisted of 18 321 pairs of drivers involved in 18 321 fatal 2-vehicle crashes between January 1, 1993, and December 31, 2016, for whom toxicological drug testing results were available. Each pair of the drivers included an initiator who was culpable of the crash (ie, whose actions or errors led to the crash) and a noninitiator who was not culpable of the crash. Initiators and noninitiators were compared within pairs as in pair-matched case-control studies.^24,27,31,32^ Excluded from the analysis were 625 317 crashes involving a single vehicle or more than 2 vehicles, 119 874 crashes involving heavy vehicles or commercial vehicles (gross vehicle weight rating ≥26 000 lbs), 137 843 crashes with missing toxicological testing results, 775 crashes in Maryland, Montana, New Mexico, and North Carolina with unreliable toxicological testing results,^5^ and 2872 two-vehicle crashes in which both drivers were culpable of crash initiation (Figure 1). The Columbia University Medical Center institutional review board deemed this study exempt from review under title 45 Code of Federal Regulations part 46. This article followed the Strengthening the Reporting of Observational Studies in Epidemiology (STROBE) reporting guideline.

### Drug Testing Assessments

Currently, 26 US states and the District of Columbia have centralized medical examiner systems, 12 have coroner systems, 3 have county or district medical examiners, and 9 have mixed medical examiner and coroner systems responsible for toxicological testing.^37^ Drug tests were conducted using blood and/or urine specimens^28^ with liquid or gas chromatography, radioimmunoassay techniques, and mass spectrometry for confirmation.^38,39^ Overall, 93.3% of the drivers included in this study had at least 1 drug test based on whole blood specimens. The FARS database records up to 3 nonalcohol drugs.^39^ If a driver tests positive for a drug metabolite, only the parent drug is recorded.^29,38^ In cases where multiple nonalcohol drugs were present, the FARS records the drugs based on the following priority order: narcotics, depressants, stimulants, marijuana, and other drugs.^29,38^ According to section 1308.2 of the US Code of Federal Regulations, prescription opioids are classified as Schedule II substances, ie, drugs with recognized and acceptable medical purposes but which have a high likelihood of abuse.^40,41^ The narcotics category in the FARS includes Schedule II drugs such as prescription opioids, illicit Schedule I drugs such as heroin, and other controlled Schedule III, IV, and V substances.^40,41^ In the present study, prescription opioids refer to injectable or oral formulations of codeine, methadone, diphenoxylate, meperidine, hydromorphine, propoxyphene, oxymorphone, morphine, oxycodone, hydrocodone, and fentanyl—fentanyl continues to be prescribed for pain management despite increasingly being used illicitly. Most prescription opioids have an elimination half-life of 1 to 4 hours and a detection window of up to 24 hours in blood samples.^42^ Blood alcohol concentrations (BACs) were measured separately from other drugs in grams per deciliter where a BAC of 0.01 g/dL or greater was considered alcohol positive.^28,29^

### Statistical Analysis

Frequency distributions of driving errors made by crash initiators were tabulated by prescription opioid testing result. The McNemar test for pair-matched data was used to compare initiators with noninitiators on binary variables such as sex, survival status, prescription opioid testing result, driving while intoxicated conviction in the past 3 years, crash within the past 3 years, license suspension within the past 3 years, and speeding conviction within the past 3 years. Pearson χ^2^ test was used to compare initiators with noninitiators on age categories and BAC levels. Currently, per se laws in the United States make it illegal to drive with a BAC level of 0.08 g/dL or higher. Therefore, BACs were assessed as a 3-category variable: less than 0.01 g/dL, 0.01 to 0.07 g/dL, and 0.08 g/dL or higher.

Prevalence of prescription opioids detected in crash initiators and noninitiators from years 1993 to 2016 was plotted and the Cochran-Armitage trend test was used to evaluate the statistical significance of changes in the prevalence of prescription opioids over time. Conditional logistic regression modeling was used to estimate odds ratios (ORs) and 95% CIs of crash initiation associated with prescription opioid use, alcohol use, and other driver characteristics. Interaction between prescription opioids and alcohol was assessed on the multiplicative and the additive scales.^43^ To assess robustness and potential biases, we conducted 3 sensitivity analyses by (1) splitting the data into 2 periods (1993-2004 and 2005-2016); (2) restricting the analysis to states that performed toxicological testing on 80% or more of all drivers involved in fatal crashes; and (3) restricting the analysis to drivers who died at the crash scene. All data analyses were performed using SAS, version 9.4 (SAS Institute Inc). Statistical significance was set at *P* < .05 for 2-tailed tests.

## Results

**Figure 2.**
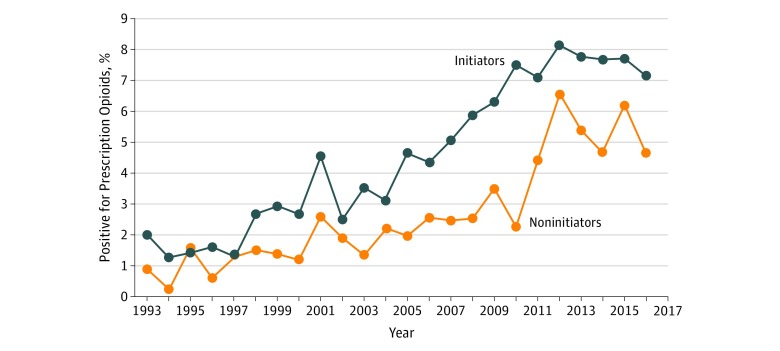
Prevalence of Prescription Opioids Detected in Drivers Involved in Fatal 2-Vehicle Crashes by Initiation Status, Fatality Analysis Reporting System, 1993-2016

The most common driving error leading to fatal 2-vehicle crashes was failure to keep in lane (7535 [41.1%]), followed by failure to yield right of way (4632 [25.3%]), and speeding (3134 [17.1%]) (Table 1). Failure to keep in lane accounted for most (502 [54.7%]) of the errors made by crash initiators who tested positive for prescription opioids (Table 1). From 1993 to 2016, the prevalence of prescription opioids detected among crash initiators increased from 2.0% to 7.1% (*P* < .001) and among noninitiators from 0.9% to 4.6% (*P* < .001) (Figure 2). Of the 1467 drivers testing positive for prescription opioids, 31.7% were positive for hydrocodone, 26.6% for morphine, 18.5% for oxycodone, 14.3% for methadone, and 8.9% for other prescription opioids. During the study period, the prevalence of alcohol use decreased from 34.2% in 1993 to 28.2% in 2016 among crash initiators and remained fairly stable at approximately 10.0% among noninitiators.

**Table 1.** Frequency Distribution of Driving Errors Involved in 18 321 Fatal 2-Vehicle Crashes, Fatality Analysis Reporting System, 1993-2016

Type of Driving Error	Crashes, No. (%)^a^		
	Prescription Opioids		Total (N = 18 321)
	Positive (n = 918)	Negative (n = 17 403)	
Failure to keep in proper lane	502 (54.7)	7033 (40.4)	7535 (41.1)
Failure to yield right of way	146 (15.9)	4486 (25.8)	4632 (25.3)
Driving too fast for conditions or in excess of posted maximum	58 (6.3)	3076 (17.7)	3134 (17.1)
Failure to obey actual traffic signs, traffic control devices, or traffic officers	100 (10.9)	2634 (15.1)	2734 (14.9)
Operating the vehicle in an erratic, reckless, careless, or negligent manner or at erratic or suddenly changing speeds	71 (7.7)	1402 (8.1)	1473 (8.0)
Driving on wrong side of road	73 (8.0)	1356 (13.9)	1429 (7.8)
Manslaughter or homicide or other assault	56 (6.1)	1022 (7.8)	1078 (5.9)
Making improper turn	14 (1.5)	522 (3.0)	556 (3.1)
Passing with insufficient distance or inadequate visibility or failing to yield to overtaking vehicle	28 (3.1)	420 (2.4)	448 (2.4)
Passing where prohibited	11 (1.2)	231 (1.3)	242 (1.3)
Any other	44 (4.8)	731 (4.2)	775 (4.2)

^a^ The total exceeds 100% as multiple driving errors could be recorded in 1 crash.

Crash initiators were more likely than noninitiators to test positive for prescription opioids (918 [5.0%] vs 549 [3.0%]; *P* < .001), alcohol (BAC ≥0.01 g/dL, 5258 [28.7%] vs 1815 [9.9%]; *P* < .001), and both substances (1.0% vs 0.3%; *P* < .001). Crash initiators were more likely than noninitiators to be younger than 35 years (9611 [52.5%] vs 6891 [37.6%] years; *P* < .001) and to be fatally injured (9341 [51.0%] vs 8401 [45.9%]; *P* < .001). Further, initiators were more likely than noninitiators to have had a positive crash history (3194 [19.3%] vs 2620 [15.8%]; *P* < .001), driving while intoxicated conviction history (894 [4.9%] vs 373 [2.1%]; *P* < .001), speeding conviction history (3955 [21.8%] vs 3264 [17.9%]; *P* < .001), and license suspension history (2987 [16.5%] vs 1572 [8.7%]; *P* < .001) in the last 3 years (Table 2).

**Table 2.** Characteristics of Drivers in Fatal 2-Vehicle Crashes by Crash Initiation Status, Fatality Analysis Reporting System, 1993-2016

Driver Characteristics	Crashes, No. (%)		*P* Value
	Initiators	Noninitiators	
Age, y			
<25	5529 (30.2)	3173 (17.3)	<.001
25-34	4082 (22.3)	3718 (20.3)	
35-44	2838 (15.4)	3601 (19.7)	
45-54	2153 (11.8)	3315 (18.1)	
55-64	1456 (8.0)	2513 (13.7)	
≥65	2263 (12.3)	2001 (10.9)	
Sex^a^			
Male	13 031 (71.1)	12 889 (70.3)	.10
Female	5286 (28.9)	5432 (29.7)	
Crash in the past 3 y^b^			
No	13 390 (80.7)	14 009 (84.2)	<.001
Yes	3194 (19.3)	2620 (15.8)	
DWI conviction in the past 3 y^c^			
No	17 247 (95.1)	17 810 (97.9)	<.001
Yes	894 (4.9)	373 (2.1)	
Speeding conviction in the past 3 y^c^			
No	14 186 (78.2)	14 919 (82.1)	<.001
Yes	3955 (21.8)	3264 (17.9)	
License suspension in the past 3 y^c^			
No	15 154 (83.5)	16 611 (91.3)	<.001
Yes	2987 (16.5)	1572 (8.7)	
BAC, g/dL			
<0.01	13 063 (71.3)	16 506 (90.1)	<.001
0.01-0.07	957 (5.2)	769 (4.2)	
≥0.08	4301 (23.5)	1046 (5.7)	
Tested positive for prescription opioids			
No	17 403 (95.0)	17 772 (97.0)	<.001
Yes	918 (5.0)	549 (3.0)	
Survival status			
Alive	8980 (49.0)	9920 (54.1)	<.001
Dead	9341 (51.0)	8401 (45.9)	

Abbreviations: BAC, blood alcohol concentration; DWI, driving while intoxicated.

^a^ Data missing for 4 drivers.

^b^ Data missing for 3429 drivers.

^c^ Data missing for 318 drivers.

Conditional logistic regression modeling revealed that prescription opioid use and alcohol use were important risk factors for fatal 2-vehicle crash initiation (Table 3). With adjustment for driver age, sex, and driving history, the estimated odds of fatal 2-vehicle crash initiation for drivers who tested positive for prescription opioids were more than twice the odds for those who tested negative (adjusted OR, 2.18; 95% CI, 1.91-2.48). Compared with drivers with BACs of 0.01 g/dL or higher, drivers with elevated BACs were at substantially increased risk of being culpable of fatal 2-vehicle crash initiation (BAC of 0.01-0.07 g/dL: adjusted OR, 1.97; 95% CI, 1.75-2.22; BAC ≥0.08 g/dL: adjusted OR, 8.20; 95% CI, 7.42-9.07 compared with BAC <0.01 g/dL) (Table 3). There was no significant interaction between prescription opioid use and BACs on the risk of fatal 2-vehicle crash initiation. Sensitivity analyses showed consistent results. When the data were divided into 2 subsets based on the year of crash (1993-2004 and 2005-2016), the estimated adjusted ORs of fatal 2-vehicle crash initiation associated with prescription opioid use were 2.36 (95% CI, 1.75-3.18) for the first period and 2.15 (95% CI, 1.86-2.50) for the second period. When the data were restricted to states that tested 80% or more of all drivers involved in fatal crashes, the estimated adjusted OR of fatal 2-vehicle crash initiation associated with prescription opioid use was 2.30 (95% CI, 1.70-3.11). Based on data for drivers who died at the crash scene, the estimated adjusted OR of fatal 2-vehicle crash initiation associated with prescription opioid use was 2.79 (95% CI, 1.71-4.54).

**Table 3.** Unadjusted and Adjusted ORs of Crash Initiation in Fatal 2-Vehicle Crashes by Prescription Opioid and BAC Testing Results and Other Driver Characteristics, Fatality Analysis Reporting System, 1993-2016

Characteristic	Crude OR (95% CI)	Adjusted OR (95% CI)^a^
Age, y		
<25	2.25 (2.10-2.41)	2.61 (2.41-2.83)
25-34	1.40 (1.30-1.49)	1.33 (1.23-1.44)
35-44	1 [Reference]	1 [Reference]
45-54	0.82 (0.76-0.88)	0.85 (0.78-0.93)
55-64	0.72 (0.67-0.79)	0.84 (0.76-0.93)
≥65	1.42 (1.31-1.53)	1.98 (1.81-2.18)
Sex		
Male	1 [Reference]	1 [Reference]
Female	0.96 (0.92-1.01)	1.18 (1.12-1.25)
Crash in the past 3 y		
No	1 [Reference]	1 [Reference]
Yes	1.34 (1.26-1.43)	1.15 (1.07-1.23)
DWI conviction in the past 3 y		
No	1 [Reference]	1 [Reference]
Yes	2.54 (2.24-2.88)	1.14 (0.96-1.34)
Speeding conviction in the past 3 y		
No	1 [Reference]	1 [Reference]
Yes	1.29 (1.22-1.35)	1.05 (0.98-1.12)
License suspension in the past 3 y		
No	1 [Reference]	1 [Reference]
Yes	2.15 (2.01-2.30)	1.55 (1.42-1.70)
BAC, g/dL		
<0.01	1 [Reference]	1 [Reference]
0.01-0.07	1.96 (1.76-2.19)	1.97 (1.75-2.22)
≥0.08	7.52 (6.87-8.24)	8.20 (7.42-9.07)
Tested positive for prescription opioids		
No	1 [Reference]	1 [Reference]
Yes	1.77 (1.58-1.98)	2.18 (1.91-2.48)
Survival status		
Alive	1 [Reference]	1 [Reference]
Died	1.16 (1.12-1.20)	1.18 (1.14-1.24)

Abbreviations: BAC, blood alcohol concentration; DWI, driving while intoxicated; OR, odds ratio.

^a^ Adjusted for all the variables listed in this table.

## Discussion

Before the opioid epidemic began in the mid-1990s, prescription opioids were rarely implicated in fatal motor vehicle crashes, detected only in approximately 1% of fatally injured drivers.^15^ In the past 2 decades, the prevalence of prescription opioids detected in fatally injured drivers has steadily increased to more than 7%.^15^ The present study provides compelling evidence that driver use of prescription opioids may double the risk of fatal 2-vehicle crash initiation, independent of demographical characteristics, driving history, and alcohol use. This finding is generally consistent with previous studies.^23,24^ A recent meta-analysis found that prescription opioid use was associated with a 47% increased risk of crash initiation.^14^ However, to our knowledge, none of the previous studies examining prescription opioid use and crash initiation used the pair-matched design; thus, they are prone to biases from spatiotemporal and environmental factors.

Results of this study suggest that the increased risk of fatal 2-vehicle crash initiation associated with prescription opioid use is owing to a large extent to increased failure to keep in proper lane. Specifically, failure to keep in proper lane accounted for more than half (54.7%) of driving errors leading to fatal 2-vehicle crashes committed by drivers testing positive for prescription opioids compared with 40.4% of the errors committed by drivers testing negative for prescription opioids. The association between prescription opioid use and increased failure to keep in proper lane was reported by Dubois et al.^27^ Failure to keep in proper lane, such as crossing the centerline, is a particularly dangerous error and might be attributable to the adverse effects of prescription opioids on alertness and lane tracking ability.^44^

Polydrug use and the potential interaction between alcohol and other drugs on driving safety are of increasing concern given that 25% of fatally injured drivers test positive for 2 or more drugs.^39^ As expected, the risk of fatal 2-vehicle crash initiation increased with BACs in a dose-response fashion. Concurrent use of prescription opioids and alcohol, however, does not seem to confer any significant interaction on the risk of fatal 2-vehicle crash initiation on either the multiplicative scale or the additive scale. Failure to detect a significant interaction effect between the 2 substances is due in part to the low prevalence of concurrent use of prescription opioids and alcohol in the study sample. The mechanisms through which alcohol and opioids can interact are complex and warrant further research. Biological interactions between alcohol and opioids are dependent on the specific drug type, amount of alcohol, and genetic differences among other factors. For example, acute alcohol consumption can enhance the effects of morphine by inhibiting cytochrome P450 3A4, the enzyme that metabolizes common opioids such as buprenorphine, oxycodone, morphine, and fentanyl.^45^ However, chronic alcohol consumption can induce cytochrome P450 3A4, leading to increased morphine metabolism and decreased analgesic effects.^45^ Alcohol and opioids may also interact in other ways.^46,47,48^ Future studies should consider including the concentration of opioids consumed to assess the dose-response effect and potential interactions with alcohol and other drugs.

Our results also indicate that drivers younger than 35 years and drivers aged 65 years or older are at heightened risk of being culpable of fatal 2-vehicle crash initiation. In addition, drivers who were involved in crashes or whose licenses were suspended in the previous 3 years are more likely than other drivers to be culpable of initiating fatal 2-vehicle crashes. Previous research has shown excess prescription opioid use among older, female, and non-Hispanic white drivers.^15,49^ As such, intervention programs, such as education and physician warnings, might target these high-risk groups. It is evident that warnings from prescribing clinicians about crash risk associated with the prescribed medication may improve drivers’ perception of crash risk.^50^ Nationally, only approximately 36% of drivers receiving prescription opioids are aware of the safety hazard posed by their medications.^17^ The Centers for Disease Control and Prevention recommend that clinicians review prescription drug monitoring program data for opioids for each patient, calculate the total morphine milligram equivalent per day, and engage each patient accordingly to avoid overprescribing and misuse.^13^ In light of the mounting evidence linking prescription opioids to motor vehicle crashes, it is sensible and necessary for clinicians to discuss driving safety with their patients while prescribing opioids.

Driving under the influence of drugs, such as opioids, is prohibited in every state. Currently, 16 states have zero-tolerance laws for all or select opioids, while Ohio and Nevada have per ser laws that specify legal cutoff concentrations for some opioids.^51^ Variation in regulations across states is due in part to inadequate research and lack of consensus on prescription opioids and driving safety. To tackle the problem of driving under the influence of drugs, law enforcement personnel are increasingly using oral fluids for quick roadside tests to screen for marijuana, opioids, and other drugs.^52,53,54^

### Limitations

This study had limitations. First, testing positive for prescription opioids indicates recent use of the medication but does not necessarily imply acute impairment resulting from the medication. However, unlike marijuana, prescription opioids have a relatively short half-life (<4 hours), which increases the likelihood of impairment given a positive test result.^42^ Second, data on opioid dosage and blood concentration are unavailable in the FARS, making it impossible to examine the dose-response relationship between prescription opioid use and the risk of fatal 2-vehicle crash initiation. Third, drug-testing data are available for only 47% of the drivers involved in fatal crashes, and drug-testing protocols may vary across states. However, restricting the analysis to fatal 2-vehicle crashes where drug-testing data were available for both drivers, complemented by the pair-matched design, should eliminate any serious information bias resulting from incomplete drug testing and variation in drug-testing protocols across states. Fourth, driving errors are used as a proxy measure of crash initiation and may not sufficiently determine culpability in some crashes. However, driving errors have been associated with crash configuration and have been widely used in crash culpability and driving safety research.^31,32,33,34,35,36^ This study assesses the association of prescription opioid use with the risk of being culpable of initiating fatal 2-vehicle crashes given being involved in these crashes. The estimated ORs reported in this study should not be used as surrogates for measuring the strength of the association between prescription opioid use and the risk of fatal crash involvement.

## Conclusions

Prescription opioids are increasingly involved in fatal motor vehicle crashes. This study provides valuable epidemiologic evidence that driver use of prescription opioids is associated with initiation of fatal 2-vehicle crashes, independent of alcohol use and demographical characteristics. While counseling patients about the risks of opioid analgesics, clinicians should take into consideration these medications’ adverse effect on driving safety.
